# 4242 Evaluating the effect of a compliant stent-graft prototype on effective stiffness in a cadaveric aorta

**DOI:** 10.1017/cts.2020.66

**Published:** 2020-07-29

**Authors:** Shannen B Kizilski, Filippo Coletti, Rumi Faizer, Victor H. Barocas

**Affiliations:** 1 University of Minnesota CTSI; 2 University of Minnesota

## Abstract

OBJECTIVES/GOALS: High aortic stiffness is associated with increased cardiovascular morbidity and mortality. The purpose of this work is to demonstrate the potential of our compliant stent-graft design to therapeutically increase aortic compliance over a standard aortic stent-graft. METHODS/STUDY POPULATION: The aorta from a human cadaver will be excised and placed into a pulse duplicator circuit. The stiffness of the system will be estimated using the pulse wave velocity (PWV), which will be calculated using the time delay between pressure measurements at proximal and distal locations in the system. Baseline measurements with the unstented aorta will be compared to two cases: (1) with a standard stent-graft placed, and (2) with our compliant stent-graft prototype in the descending thoracic aorta. PWV is calculated as the distance between the pressure sensors divided by the time delay. Faster PWV is associated with a stiffer vessel, or lower aortic compliance. RESULTS/ANTICIPATED RESULTS: Prior work *in vitro* showed that the compliant stent-graft reduced peak and pulse pressures compared a standard, rigid stent-graft. We also expect the compliant device to exhibit lower PWV compared to a rigid stent-graft. Depending on the aortic tissue stiffness, the compliant stent-graft could raise or lower PWV compared to no stent. Mean pressure in the compliant case is likely to be slightly higher than the other two cases because the compliant stent-graft’s narrower lumen increases flow resistance. Although mean pressure will be higher, peak pressure should be lower than in the standard stent-graft because the added compliance decreases overall pressure swing between systole and diastole. DISCUSSION/SIGNIFICANCE OF IMPACT: Lower PWV in the compliant stent-graft over the standard stent-graft will indicate its potential to therapeutically lower aortic stiffness in patients needing aortic stenting. Positive outcomes from this study will be a step toward the eventual translation of a compliant stent-graft to clinical use.

